# Supplementary material for: Association between self-reported and proxy informant Health Quality of life of older adults with the use of antipsychotic drugs in nursing homes. A cross-sectional study
Source: PLoS One. 2025 Jan 7;20(1):e0311823. doi: 10.1371/journal.pone.0311823 (PMC11706377; doi:10.1371/journal.pone.0311823)
Supplement: Attachment — Submitted filename: Response to Reviewers.docx [file pone.0311823.s003.docx]

5. Review Comments to the Author

***Reviewer #1:*** *Your study highlights the complexity of managing antipsychotic use in nursing homes, emphasizing the need to consider diverse perspectives to improve patient care. Recognizing the multifaceted nature of quality of life and using varied assessment tools can provide a more comprehensive understanding of antipsychotic medication effects. The focus on this complexity is essential for advancing the field and enhancing patient care. Additionally, your study offers valuable insights into the impact of antipsychotic medications on nursing home residents' quality of life, highlighting both strengths and limitations. Implementing recommendations for future research can contribute to improved care for individuals in nursing homes.*

**Authors comment:** We thank the reviewer for her/his encouraging comments and its interest on our work.

***Reviewer #2:*** *Thank you for the opportunity to review the manuscript. The manuscript is well written, the methodology is sound, and the findings are clearly articulated, providing valuable insights into the potential use of antipsychotic drugs on the quality of life of older adults in nursing homes. However, there are a few areas that need revisions for clarity and completeness.

1. Introduction : Emphasize the importance of the study’s findings for clinical practice and policy-making in nursing homes, explaining how the results can influence the management of antipsychotic use.*

**Authors comment:** First and foremost, we would like to thank the reviewer for their comments, all of which we have taken into consideration. We agree with the reviewer’s comment 1. We have added this point in the introduction section:

“There is a disparity in perception between clinicians working in nursing homes who regularly prescribe antipsychotics, patients’ views on the usefulness of their treatment, and health authorities advocating for substantial restrictions on this class of medication. Nevertheless, the shared objective of all parties is to maintain or improve the quality of life of the residents. The study of the association between quality of life and antipsychotic use could facilitate the development of shared decision-making processes, in which patients and clinicians collaborate to make treatment choices based on the patients’ preferences and the best available evidence.”

With the following reference:

Haugom EW, Benth JŠ, Stensrud B, Ruud T, Clausen T, Landheim AS. Shared decision making and associated factors among patients with psychotic disorders: a cross-sectional study. BMC Psychiatry. 2023;23(1):747. Published 2023 Oct 13. doi:10.1186/s12888-023-05257-y

*2. Methods : Provide a rationale for the selection of EQ-5D-3L and NPI-R measurement tools, explaining why they are appropriate for assessing HRQoL and psychotic symptoms in this study population.*

**Authors comment:** We agree with the reviewer’s comment. We have to justify the use of these two tools in the method section. Considering our target population (older adults in nursing homes, with 74% suffering from major cognitive disorders), we consider that EQ5D-3L and NPI are both valid and relevant for our study. We have added this in the method section:

“The EQ-5D scale is a globally recognized instrument for assessing quality of life and has been extensively utilized across various countries. The EQ-5D-3L questionnaire is also considered a valuable predictor of mortality and initial hospitalization in older populations.” with the following reference:

Cavrini G, Broccoli S, Puccini A, et al. EQ-5D as a predictor of mortality and hospitalization in elderly people. Qual Life Res. 2012;21:269–80.

And for the NPI-R:

“The Neuropsychiatric Inventory (NPI) is one of the most widely utilized scales for assessing symptoms in individuals with dementia and other neurological disorders. Originally developed for populations with dementia, the NPI has also been employed in evaluating patients with psychotic, affective, and other neurological conditions, including Parkinson’s disease.” with the reference:

Lai CK (2014) The merits and problems of Neuropsychiatric Inventory as an assessment tool in people with dementia and other neurological disorders. *ClinIntervAging* 9,1051–1061

*3. Describe the data collection process in detail. Add details about the timing (e.g., frequency of assessments, duration of each session) and methods (structured interviews, use of standardized questionnaires) used to collect data from participants and proxies.*

**Authors comment:** We agree with the reviewer’s comment. We have added in the method section:

“Healthcare professionals, including experienced geriatricians and clinical research nurses, conducted interviews with the participants and their professional caregivers. The participant interview included a physical examination (assessing grip strength, gait speed, and blood pressure), a cognitive evaluation (MMSE test), and a psychological assessment incorporating the EQ-5D-3L scale. Professional caregivers provided detailed information on various aspects of the participants, including sociodemographic data, medical history, medication, nutritional status, level of activity, degree of dependence (ADL), and the EQ-5D-3L scale. The estimated completion time was 30 minutes but varied according to the participant’s physical and cognitive condition.”

*4. Expand the ethics section to include details on how data confidentiality was maintained and any measures taken to ensure the welfare of participants during the study.*

**Authors comment:** We agree with the reviewer’s comment. We have added this point in the ethics section:

**“**Participants received an informational leaflet outlining the key aspects of the study. Participation was entirely voluntary, and individuals had the option to decline participation or withdraw at any point without facing any negative consequences. Considering the high prevalence of cognitive impairment among nursing home residents, the on-site investigator took care to ensure that participants fully comprehended the implications of their involvement. In instances where a participant was under legal guardianship and/or unable to understand the study, non-opposition was obtained from the legal guardian or a designated contact person. All data were anonymized.”

*5. Results : Clearly indicate the statistical significance of the results within the text by mentioning p-values for all relevant comparisons and analyses. This will help readers quickly understand which results are statistically significant without having to refer to the tables.*

**Authors comment:** We agree with the reviewer’s comment. We have incorporated the statistical results into the text. Indeed, both the text and tables should be able to stand alone, allowing the reader to understand them independently.

*6. Identify and discuss any potential confounding variables (such as severity of cognitive impairment, presence of comorbid conditions, medication adherence, and nursing home environment) that could have impacted the study’s outcomes.*

**Authors comment:** We agree with the reviewer’s comment and added several limits to our study, notably medication adherence, and the impact of other NPS on HrQOL. The severity of cognitive impairment was a limitation we had previously identified.
